# Non-interactive zero-knowledge proof scheme from RLWE-based key exchange

**DOI:** 10.1371/journal.pone.0256372

**Published:** 2021-08-20

**Authors:** Shaofen Xie, Wang Yao, Faguo Wu, Zhiming Zheng

**Affiliations:** 1 School of Mathematical Sciences, Beihang University, and Key Laboratory of Mathematics, Informatics and Behavioral Semantics, Ministry of Education, Beijing, China; 2 Institute of Artificial Intelligence, Beihang University, and Key Laboratory of Mathematics, Informatics and Behavioral Semantics, Ministry of Education, Beijing, China; 3 Research Institute for Frontier Science, Beihang University, and Key Laboratory of Mathematics, Informatics and Behavioral Semantics, Ministry of Education, Beijing, China; 4 Peng Cheng Laboratory, Shenzhen, Guangdong, China; 5 Beijing Advanced Innovation Center for Big Data and Brain Computing, Beihang University, Beijing, China; Victoria University, AUSTRALIA

## Abstract

Lattice-based non-interactive zero-knowledge proof has been widely used in one-way communication and can be effectively applied to resist quantum attacks. However, lattice-based non-interactive zero-knowledge proof schemes have long faced and paid more attention to some efficiency issues, such as proof size and verification time. In this paper, we propose the non-interactive zero-knowledge proof schemes from RLWE-based key exchange by making use of the Hash function and public-key encryption. We then show how to apply the proposed schemes to achieve the fixed proof size and rapid public verification. Compared with previous approaches, our schemes can realize better effectiveness in proof size and verification time. In addition, the proposed schemes are secure from completeness, soundness, and zero-knowledge.

## Introduction

With the development of information networks, the concern about privacy of personal data is growing. Users tend to communicate on the Internet without revealing individual data [1–7], such as users’ passwords, personal assets information, personal health condition, and so on. According to cryptography theory, the zero-knowledge(ZK) proof [8] is an essential technique for preserving information protection. In the 1880s, Goldwasser et al. proposed the interactive zero-knowledge proof for the first time. Subsequently, Blum et al. [9] first proposed the non-interactive zero-knowledge proof, which is characterized by only one-time communication between the prover and the verifier. Andre et al. [10] designed the protocol which was based on the public-key cryptosystem RSA-encryption for the proof of ownership. Siamak et al. pointed that the significance of credential ownership proofs and gave the proof by the RSA signature scheme [11]. Over the past three decades, extensive research has been conducted on zero-knowledge proofs in terms of algorithm safety and operational efficiency [12–14]. And it is used in various fields such as authentication, ownership, etc. [15–17].

Since the non-interactive zero-knowledge proof is suitable for offline operations, it will have more application scenarios. Usually, there are two methods for interactive zero-knowledge proof to achieve non-interactive zero-knowledge proof: Fiat-Shamir heuristic and CRS(Common Reference Strings) model. For the Fiat-Shamir heuristic, it demands to include a random oracle to generate a uniformly random output. For the CRS model, it needs to rely on a trusted third-party or secure multi-party computing to obtain the common reference string. There has been a series of corresponding studies using the Fiat-Shamir heuristic. Lindell et al. [18] and Ciampi et al. [19] have redesigned the scheme to improve security and efficiency based on Fiat-Shamir transform. With the rapid application of cryptocurrency, after Groth [20] proposed the scheme of constant-size proofs, the first zero-knowledge succinct non-interactive argument of knowledge (ZK-SNARK) based on the assumption of a trusted third-party has also been widely studied. The zero-knowledge proof schemes of traditional encryption have been widely used. And they have made great contributions in efficiency, security, and so on. However, researchers have paid less attention to zero-knowledge proof schemes that can resist quantum attacks.

With the surprising development of quantum computers, a series of post-quantum cryptographic algorithms have been studied [21]. The lattice-based cryptographic algorithm could effectively resist quantum attacks. After Ajtai et al. [22] introduced lattice cryptography and gave strict proof from the average case to the worst case for the first time. Due to the security and applicability of lattice-based cryptographic algorithms, extensive research has been conducted. In 2010, RLWE(Ring Learning with Errors), proposed by Lyubashevsky et al. [23], significantly shortened the key length and improved the efficiency of the signature. Lattice-based cryptography theories have also begun extensive research and application. Subsequently, lattice-based zero-knowledge proofs have begun to develop gradually.

### Related works

In 2012, Vadim Lyubashevsky used Fiat-Shamir with Aborts technology to design a zero-knowledge proof scheme [24], which proves that *s* satisfies *As* = *t* **mod** *q* without revealing the secret value *s*. The signature size is 16500 bits which can be modified to shorten using the compression technique, where the scheme relies on SIS(Small Integer Solution) problem and LWE(Learning with Errors) problem. The scheme was then applied and extended by Vadim Lyubashevsky and Gregory Neven to a ciphertext-verifiable scheme [25]. At the same time, Fabrice Benhamouda et al. [26] used zero-knowledge to prove that the secret value *s* satisfies *p* = *as* + *e* and was used in group signature authentication to protect the anonymity of group members. However, the security is based on a lattice-based assumption and the discrete-logarithm problem. Then we should consider how to construct an effective zero-knowledge proof system in terms of the proof size and the security to promote its application.

Most studies on commitment schemes are quite effective practice for zero-knowledge proof, such as [27–33]. Fabrice Benhamouda et al. [27] constructed a simple efficient string commitment scheme based on RLWE and the zero-knowledge proofs of knowledge for linear relations and multiplicative relations, where the communication complexity is O(Mnlog(q)). In order to achieve a negligible soundness error, the protocol demands run *M* rounds. Vadim Lyubashevsky et al. [28] proposed splitting the polynomial into multiple factors. This method can improve the computation and more information will be hidden. Baum et al. [29] constructed a novel zero-knowledge proof of preimages scheme which would become a tool against malicious adversaries. Muhammed et al. [30] used the one-shot proof technique for non-linear polynomial relations to improve computing and communication performance. Baum et al. designed the zero-knowledge argument based on the short integer solution assumption for specific languages. The communication complexity of this scheme is $\sqrt{mlog(m)}$, where *m* is the number of gates [31]. The proof size of Bootle et al. proposed scheme [32] is better than that of Stern type proof, but the efficiency of verification time still needs to be improved. Baum et al. [33] constructed a practical zero-knowledge proof of opening knowledge which is an improvement of the scheme of Fabrice Benhamouda et al. [27]. It has shown that prover does not need to send more proof for part of the calculation process. Obviously, the disadvantage of all these previous commitment schemes is that the commitments also consume calculation and communication in the interactive. Meanwhile, considering that the interactive property and the proof size, the commitment schemes are no longer suitable for the publicly-verifiable scheme. We would adopt non-interactive zero-knowledge proof to design a publicly-verifiable scheme with more short proof size and verification time.

Subsequently, related research has also improved in terms of efficiency and safety [34, 35]. Among them, Rafael et al. [34] proposed a scheme that could reduce the proof size. The main idea of this method is to reduce the number of equations in the protocol by increasing the running time of the proof. It is also an interactive scheme that is different from the non-interactive that we would discuss. The non-interactive zero-knowledge proof of LWE based on the SSP (Square Span Programs) in ZK-SNARKs was proposed by Rosario Gennaro et al. [35] and is considered post-quantum secure, and which would be used in the anonymous cryptocurrency Zerocash. The proof size of this scheme is constant which just consists of 5 LWE encodings. Rosario Gennaro et. al. mainly focused on designated-verifier proofs, but we would study the publicly-verifiable proofs. Ding has used the signal function from the RLWE key exchange to design an effective interactive zero-knowledge authentication protocol [36]. They pointed out that the design of the RLWE-based non-interactive zero-knowledge authentication scheme will be future work. Based on the above comparative analysis, we would construct the new non-interactive zero-knowledge proof protocols that can prove ownership with small proof size and high efficiency.

### Motivation

Your protected private data can be used in a variety of scenarios, for example, you have a private key, or you have enough money to pay for the transaction, or you know the solution to the problem. In other words, prover should generate the proof for the verifier to prove the secret value for ownership, account balance, Sudoku, etc. Because of the convenience of one-time communication, we study the lattice-based non-interactive zero-knowledge proof against quantum attacks. Considering the wide application of scenarios, the scheme is extended from designated-verifier verification to publicly-verifiable. Because we do not completely trust the trusted third-party, and we want nobody to know our secret value. Therefore, we introduce the semi-trusted third-party assumption, that is, the third-party can only prove the secret value but know nothing about it. This assumption can further enhance the protection of the secret and ensure the security of the scheme.

### Contributions

The main contributions in this paper are as follows:

1. We design a non-interactive zero-knowledge proof scheme to ensure that the proof size is constant for the designated-verifier. Then, we prove that this scheme is secure in terms of completeness, soundness, and zero-knowledge.
2. In order to satisfy the fixed proof size and improve verification efficiency, we combine public-key encryption with a semi-trusted third-party assumption to construct a publicly-verifiable scheme and prove the security of the scheme.
3. Compared with the previous scheme with efficiency advantage, we achieve better effectiveness schemes in zero-knowledge proof size and verification time.

### Organization

The rest of this paper is organized as follows. In Section 2, we show the essential preliminary for the schemes. In Section 3, we construct a zero-knowledge proof scheme based on key-exchange in designated-verifier and provide the security analysis of the scheme. In Section 4, we construct the non-interactive zero-knowledge proof for publicly-verifiable and also give the security analysis. In Section 5, we compare the efficiency of our schemes with the previous existing protocols. In Section 6, we give a summary of this paper.

## Preliminaries

### Notations

Throughout the paper, following notations would be used in Table 1.

**Table 1:** Description of notations.

Notation	Description
*n*	security parameter, it’s a power of 2
*q*	a positive integer
$Z_{q}$	the ring Z/qZ
*x*^*n*^ + 1	irreducible polynomial
*R*_*q*_	the ring $Z_{q}\left[x\right]/\left(x^{n}+1\right)$
${\left\{a_{i}\right\}}_{i=0}^{n-1}$	any polynomial *a* ∈*R*_*q*_, using the coefficient vector {*a*_0_, *a*_1_, ⋯, *a*_*n*−1_} in $Z_{q}$ to represent *a*
*M* ^*T*^	the transpose of the matrix *M*
‖*s*‖_∞_	*max*|*s*_*i*_| for a vector *s*
‖*s*‖	‖*s*‖_*q*_, ${\left(\sum_{i=0}^{n-1}|v_{i}{|}^{q}\right)}^{1/q}$ for a vector *s*
D	the discrete Gaussian distribution on lattice

### Non-interactive zero-knowledge proof

**Definition 1***Non-Interactive Zero-Knowledge Proof: (P,V) is a non-Interactive zero-knowledge proof system for a language L, where s is secret value, should satisfy the following properties* [9]:

#### Completeness

*If the statement is true, the honest verifier will be convinced of this fact by an honest prover*. *That means, for*∀x∈L, *if the prover has the secret value s, then*

$$Pr\;\left[\pi \leftarrow P\left(x,s\right):V\left(x,\pi \right)=1\right]\geq 1-2^{-n}.$$

#### Soundness

*If the statement is false, none prover as the adversary can convince the honest verifier that it is true, except with negligible probability*. *That means, for*∀x∉L, *if the prover has no secret value s, then*

$$Pr\;\left[\pi \leftarrow P\left(x\right):V\left(x,\pi \right)=1\right]\leq 2^{-n}.$$

#### Zero-knowledge

*If the statement is true, the verifier will learn nothing about the secret but the fact that the statement is true*.

*That is, there is a polynomial-time simulator, for all*x∈L*and secret value s, the following two distributions are computationally indistinguishable, whereExp*_*ZK*−*real*_ = 1 *represents the algorithm of the real system and* [*Exp*_*ZK*−*sim*_ = 1] *represents the algorithm of the simulation system*.

$$\|Pr\;\left[Exp_{ZK-real}=1\right]-Pr\;\left[Exp_{ZK-sim}=1\right]\;\|\leq 2^{-n}.$$

#### Non-interactive

*The prover only has one-way communication to the verifiers during the proof phase*.

### Ring learning with error

Here, we recall informally the Ring Learning with Error assumption, which is a classical hard problem on lattice defined by Regev [37]. And the security of our quantum-resistant schemes rely on the hardness assumption that has been previously used in the literature as follows.

**Definition 2***Discrete Gaussian: For*$\forall \sigma >0,x\in Z^{N}$, *the center of Discrete Gaussian Distribution c, and defined*$\rho_{\sigma ,c}\left(x\right)={\left(\frac{1}{\sigma }\right)}^{n}e^{\frac{-\pi {\left\|x-c\right\|}^{2}}{\sigma^{2}}}$*as the N-dimensional Gaussian function: Then the Discrete Gaussian Distribution can be defined as*$D_{L,\sigma ,c}=\frac{\rho_{\sigma ,c}\left(x\right)}{\rho_{\sigma ,c}\left(L\right)}$.

**Lemma 1** ([38]). *Let* $R=Z\left[x\right]/\left(x^{n}+1\right)$, *for any* *a*, *b* ∈ *R*, *we have* $\left\|a\cdot b\right\|\leq \sqrt{n}\cdot \left\|a\right\|\cdot \left\|b\right\|$ *and* ‖*a* ⋅ *b*‖_∞_ ≤ ‖*a*‖_∞_ ⋅ ‖*b*‖_∞_.

**Lemma 2** ([39]). *For any* $\sigma \geq \omega (\sqrt{log(n)})$, *then we have*

$$\underset{x\leftarrow D_{L,\sigma ,c}}{Pr}\;[\|x\|>\sigma \sqrt{n}]\leq 2^{-n}.$$

**Definition 3** *RLWE Problem* [36, 37]: *Let* n∈Z *be a power of 2 and q be positive integers, and let* *χ*_*α*_ *be the Discrete Gaussian distribution onR*_*q*_. *For a uniformly chosen element s of the polynomial ring R*_*q*_, *let* $A_{s,\chi_{\alpha }}$ *be the distribution of the pair* (*a*, *as* + 2*e*) ∈ *R*_*q*_ × *R*_*q*_, *where* *a* ← *R*_*q*_ *is uniformly chosen and e* ← *χ*_*α*_ *is independent of a*. *Then the* $A_{s,\chi_{\alpha }}$ *is computationally indistinguishable from the uniform distribution on R*_*q*_ × *R*_*q*_ *for any probabilistic polynomial time(PPT)*.

**Assumption 1** [36] *The security of our schemes rely on the Ring-LWE assumption stating that the distribution of* (*h*_*i*_, *h*_*i*_ *s* + *e*_*i*_), *where h*_*i*_ *is random in R*_*q*_ *and* *s*, *e*_*i*_ *are small polynomials, is indistinguishable from uniform*. *The search version of Ring-LWE is to modify the above definition by requiring the PPT algorithm to find s rather than distinguish the two distributions*.

### Signal function

Given $Z_{q}$ is $\left\{-\frac{q-1}{2},\cdots ,\frac{q-1}{2}\right\}$, we introduce the signal function [38] to eliminate the errors caused by Mod_2_ function in *R*_*q*_.

**Definition 4***Signal Function Let*$E_{1}=\left[-\left\lfloor \frac{q}{4}\right\rfloor \;,\left\lfloor \frac{q}{4}\right\rceil \;\right]$*and*$E_{2}=\left[-\left\lfloor \frac{q}{4}\right\rfloor \;+1,\left\lfloor \frac{q}{4}\right\rceil +1\right]$, *then the signal function*$\text{Char}:Z_{q}\to \left\{0,1\right\}$*as the following*:

$$\text{Char}\left(x\right)=\{\begin{array}{ccc} 0 & & x\in E_{1}\;\text{or}\;x\in E_{2}\\ 1 & & \text{otherwise} \end{array}.$$

**Definition 5***The function*${\text{Mod}}_{2}:Z_{q}\times \left\{0,1\right\}\to \left\{0,1\right\}$*was defined as*

$${\text{Mod}}_{2}\left(x,a\right)=\left(x+a\cdot \frac{q-1}{2}\right)\;\;{\text{Mod}}_{q}\;{\text{Mod}}_{2}.$$

**Lemma 3** ([40]). *Let q* > 8 *and be an odd prime*, $x,y\in Z_{q}$ *such that* ${\left\|x-y\right\|}_{\infty }<\frac{q}{4}$ *and* *a* = Char(*x*). *Then*

$${\text{Mod}}_{2}\left(x,a\right)={\text{Mod}}_{2}\left(y,a\right).$$

**Proof 0.1** *Known* ${\left\|x-y\right\|}_{\infty }<\frac{q}{4}$, *let* *x* = *y* + 2*ε*, $\left|2\varepsilon \right|\leq \frac{q}{4}-2$, *Note that* $\left(x+a\cdot \frac{q-1}{2}\right){\text{mod}}_{q}=\left(y+a\cdot \frac{q-1}{2}+2\varepsilon \right){\text{mod}}_{q}$. *From the Char function as we defined*, $|y+a\cdot \frac{q-1}{2}|<\frac{q}{4}+1$, *because* $|y+a\cdot \frac{q-1}{2}+2\varepsilon |\leq |y+a\cdot \frac{q-1}{2}\left|+|2\varepsilon \right|\leq \frac{q}{2}-1$, $\left(y+a\cdot \frac{q-1}{2}+2\varepsilon \right){\text{mod}}_{q}=\left(y+a\cdot \frac{q-1}{2}\right){\text{mod}}_{q}++2\varepsilon$, *Perform* mod_2_ *on both sides of the above equation, then* ${\text{mod}}_{2}\left(x,a\right)=\left(x+a\cdot \frac{q-1}{2}\right){\text{mod}}_{q}{\text{mod}}_{2}=\left(y+a\cdot \frac{q-1}{2}\right){\text{mod}}_{q}{\text{mod}}_{2}={\text{mod}}_{2}\left(y,a\right)$.

Then, signal function and mod_2_ function were extended to polynomial ring *x* ∈ *R*_*q*_ by applying them for each coefficient $x_{i}\in Z_{q}$. Clearly, the result in Lemma 3 still holds when extending to polynomial ring elements. In the following, we use the extend signal function and mod_2_ function in the polynomial ring $R_{q}^{n\times n}$.

As far as we know, Ding et al. [41] in 2017 pointed out that signal function may leak the secret key when RLWE public-key is reused for the long term. But it does not mean that the signal function cannot be used forever. Gao et al. [42] used a key reuse mode by adding the user ID and a fresh public error against this attack in 2018.

## Designated-verifier non-interactive zero-knowledge Proof based on RLWE key exchange

### DVNIZK scheme

Firstly, we describe the designated-verifier non-interactive zero-knowledge proof scheme (DVNIZK) which is based on RLWE key exchange. As we know, only the designated-verifier can effectively check the proof in the DVNIZK scheme. This scheme is just a one-time proof. That is to say, a new session will be built for the different designated-verifier. At the same time, the prover will generate fresh errors for different verifiers. Therefore, this scheme can against signal function leakage attacks.

**Fig 1:**
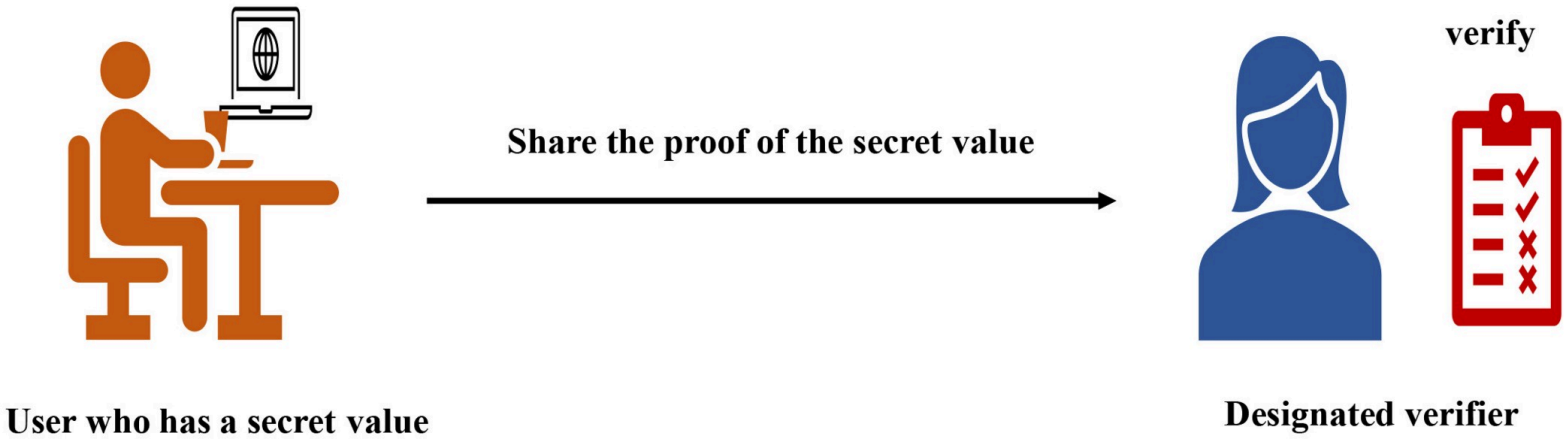
The designated-verifier scheme architecture.

This scheme involves two objects: prover and verifier. Prover generates the proof for the verifier to prove the user of secret value for ownership, account balance, Sudoku, etc, which is illustrated in Fig 1.

**Fig 2:**
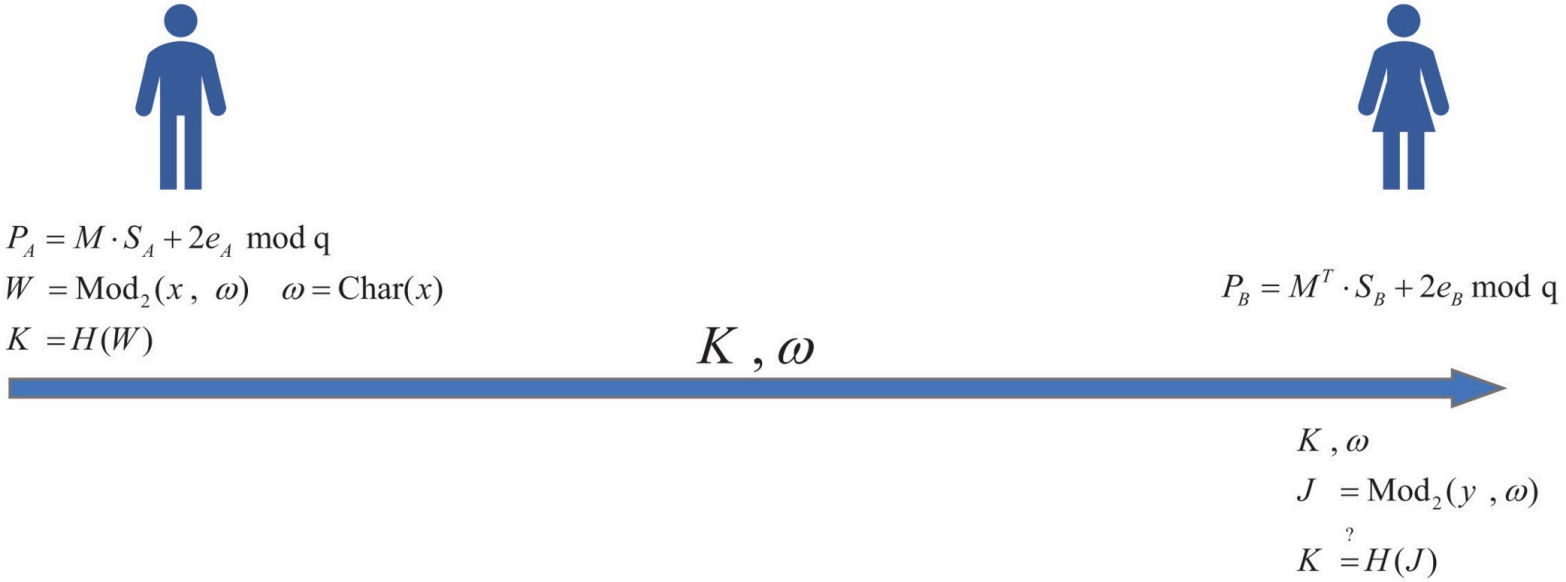
Designated-verifier non-interactive zero-knowledge based on RLWE key exchange.

In the first scheme, we provide the security model for the designated-verifier non-interactive zero-knowledge proof using the random oracle model, similar to the security model proposed in [36]. Our first scheme is composed of three algorithms: Setup, Proof, Verify. Specifically, we demonstrate it as follows, shown in Fig 2.

1.Setup:

- The public parameters *q*, *n*, *α* be generated, where *q* > 8 is prime.
- Let *M* be a uniformly random matrix $M\leftarrow R_{q}^{n\times n}$.
- Let *H*: {0, 1}^*n* × *n*^ → {0, 1}^*n*^ be a random oracle.
- P has *P*_*A*_ = *M* ⋅ *S*_*A*_ + 2*e*_*A*_ mod *q* where *S*_*A*_ is secret information and *S*_*A*_, *e*_*A*_ ← *χ*_*α*_ are *n* × *n* dimensional. Namely, *S*_*A*_ consists of *n* elements in a polynomial ring (i.e. *S*_*A*_ = (*S*_1_, *S*_2_, ⋯, *S*_*n*_)^*T*^, where *S*_*i*_ = (*s*_*i*1_, *s*_*i*2_, ⋯, *s*_*in*_)∈*R*_*q*_, $s_{ij}\in Z_{q}$, which *i* and *j* range from 1 to *n*). *s*_*ij*_ is sampled from the Discrete Gaussian distribution with parameter *σ*. Clearly, *S*_*A*_ can be expressed as a *n* × *n* dimensional matrix. And *e*_*A*_ is the same as above *S*_*A*_. It is stated here that all the multiplication operations of the elements in the polynomial ring must undergo modulo *q* and modulo the irreducible polynomial *x*^*n*^ + 1 operations.
- V has *P*_*B*_ = *M* ⋅ *S*_*B*_ + 2*e*_*B*_ mod *q* where *S*_*B*_, *e*_*B*_ ← *χ*_*α*_ are also *n* × *n* dimensional. *S*_*B*_ and *e*_*B*_ are also same as above *S*_*A*_.

2.Proof:

- Let $x=S_{A}^{T}\cdot P_{B}$, P computes *ω* = Char(*x*) and *W* ← Mod_2_(*x*, *ω*) as the zero-knowledge proof.
- P computes *K* = *H*(*W*).
- P sends *K*, *ω* to V.

3.Verify:

- Upon receiving *K*, *ω*, only verifier could do the following:
- Let $y=P_{A}^{T}\cdot S_{B}$, V computes *J* ← Mod_2_(*y*, *ω*) firstly.
- V checks if $K\overset{?}{=}H\left(J\right)$. Accept if *K* = *H*(*J*), otherwise reject.

### Security analysis

Security is the most important factor of the zero-knowledge proof schemes. The first scheme for designated-verifier only can be used as the one-time proof. It satisfies the properties of the zero-knowledge proof: Completeness, Soundness, Zero-knowledge. We will supply a simple security analysis in the following.

**Lemma 4***Letq* > 16*σ*^2^ *n*^*α*3/2^, *then* Mod_2_(*x*, *ω*) = Mod_2_(*y*, *ω*), *except with negligible probability*.

**Proof 0.2***Firstly, we know that*$x=S_{A}^{T}\cdot P_{B}$*and*$y=P_{A}^{T}\cdot S_{B}$.

*Considering that*$x,y\in R_{q}^{n\times n}$, *then we just show that*$\|x_{i,j}-y_{i,j}{\|}_{\infty }<\frac{q}{4}$*by Lemma 3, wherei*, *j* ∈ {1, ⋯, *q*}. *After simplification, combining with Lemma 1 and Lemma 2, we have that*

$$\|x_{i,j}-y_{i,j}\|=\|2\left(S_{A_{i,j}}^{T}\cdot e_{B_{i,j}}-e_{A_{i,j}}^{T}\cdot S_{B_{i,j}}\right)\|<4\sigma^{2}n^{3/2}$$

*By lemma 3, we obtain* $\|x_{i,j}-y_{i,j}{\|}_{\infty }<4\sigma^{2}n^{3/2}<\frac{q}{4}$.

*Then, q* > 16*σ*^2^ *n*^*α*3/2^ *with probability* 1 − 2^−*n*^.

#### 1) Completeness

The form of the proof is shown as follows:

As we know *K* = *H*(*W*), where *W* = Mod_2_(*x*, *ω*).

The verifier owns *S*_*B*_, *P*_*A*_, *K*, *ω* after receiving the proof, it will follow from straightforward calculations *H*(*J*) where *J* = Mod_2_(*y*, *ω*).

If *S*_*A*_ is true, by the lemma 4, Mod_2_(*x*, *ω*) = Mod_2_(*y*, *ω*). That means *W* = *J*. Finally, *K* = *H*(*W*) will be equal to *H*(*J*). Then the verifier will be convinced that the prover owns the secret.

#### 2) Soundness

Assuming that P would be a adversary and as an oracle that generate the valid proof for the honest verifier with non-negligible probability in the following.

***Game*_0_**: this game is the original scheme. According to the scheme, *P*_*A*_ = *M* ⋅ *S*_*A*_ + 2*e*_*A*_ mod *q*, *P*_*B*_ = *M*^*T*^ ⋅ *S*_*B*_ + 2*e*_*B*_ mod *q*, then *W*, *ω*, *K*, *J* is generated, and output *b*_*d*_ which is the determination of the verifier. Among which *b*_*d*_ = 1 means the verifier accepts and *b*_*d*_ = 0 respects he rejects.

***Game*_1_**: this game is equal to ***Game*_0_** except that *P*_*A*_ is chosen uniformly. A cannot generate the valid proofs for the chosen uniform *P*_*A*_ with negligible probability.

**Lemma 5***If prover as the adversary*A*who could generate a valid proof for P*_*A*_ = *M* ⋅ *S*_*A*_ + 2*e*_*A*_ *mod* *q* *without the secret of S*_*A*_ *to convince the honest verifier with non-negligible probability, then there is a distinguisher for* *P*_*A*_ *from uniform with the same probability*.

**Proof 0.3***Assuming that*B*be a RLWE distinguisher*. *Considering that interaction between*A*and*B*as the role of honest verifier*. *When*B*sends the challenge* (*M*, *P*_*A*_) *from the RLWE challenger to* A, A *would do the following*. *If* *P*_*A*_ = *M* ⋅ *S*_*A*_ + 2*e*_*A*_ *mod* *q*, *the interaction between* A *and* B *is equal to* *Game*_0_, *then* B *outputs 1*. *If P*_*A*_ *is chosen uniformly, the interaction between* A *and* B *is equal to* *Game*_1_, *then* B *outputs 0*.

*Suppose*A*can generate a valid proof for the chosen uniform P*_*A*_*with non-negligible probability, then*B*would not be successful from the above defined*. *That means the adversary can solve the search version of the Ring-LWE problem with the same probability*. *Suppose*A*can generate K and ω which are accepted by the honest verifier for P*_*A*_*without the secret of S*_*A*_. *In our first scheme, the crucial verify are J* = Mod_2_(*y*, *ω*). *It means that the valid proof only could be obtained by guessed or correctly calculated* Char(*y*) *and* Mod_2_(*y*, Char(*y*)).

*On the one hand, the valid proof that the probability of being brutally guessed is negligible. On the other hand, the adversary wants to calculate* Char(*y*) *and* Mod_2_(*y*, Char(*y*)) *correctly*. *Considering that this scheme is used as a one-time proof, there is no sign function leakage attack that makes the secret value leak*. *Therefore, in order to calculate the correct* Char(*y*) *and* Mod_2_(*y*, Char(*y*)), A *needs to know the secret value S*_*B*_. *However, if* A *knows the secret value S*_*B*_, *it means that it can solve the search version of the Ring-LWE problem*. *In fact, the search version of Ring-LWE is to modify the above definition by requiring the PPT algorithm to find s rather than distinguish the two distributions in Assumption 1*. *Therefore, the adversary can generate a valid proof for the chosen uniform P*_*A*_ *and would be accepted by the designated-verifier with the same probability which can solve the search version of the Ring-LWE problem*.

In summary, in DVNIZK-KE Scheme, the adversary cannot convince the verifier to believe him with negligible probability without knowing the secret value. Then the soundness error of our is negligible.

#### 3) Honest-verifier zero-knowledge

**Lemma 6***Honest-verifier Zero-knowledge* [26] *means that there be a probabilistic polynomial-time simulator taking P*_*A*_ *and* $S_{B}^{{}^{\prime }}$ *as input, that outputs* (*K*′, *ω*′) *is indistinguishable from an accepting protocol transcript generated by a real scheme run*.

**Proof 0.4***Then we assume a probabilistic polynomial-time simulator*S*would access to the random oracle*H. *Then we construct Exp*_*ZK*−*sim*_*to make zero-knowledge proof in the random oracle model*. *Let Exp*_*ZK*−*real*_ = 1 *denotes the output of the interactive scheme between the real prover and the designated-verifier*. *Exp*_*ZK*−*sim*_ = 1 *means the simulator’s output*. *The simulator* S *for our first scheme described in* Fig 1 *is constructed: query the random oracle* H *with input* $S_{B}^{{}^{\prime }}$ *and output* $K^{{}^{\prime }}=H\left(W^{{}^{\prime }}\right)=H\left({\text{Mod}}_{2}\left(P_{A}^{T}\cdot S_{B}^{{}^{\prime }}\;,\;\omega^{{}^{\prime }}\right)\right)$ *which* $\omega^{{}^{\prime }}=\text{Char}\left(P_{A}^{T}\cdot S_{B}^{{}^{\prime }}\right)$.

*According to the construction, we claim that the outputs from a probabilistic polynomial-time simulator are indistinguishable from an accepting protocol transcript generated by a real scheme run*.

Considering the scheme of expandability, the prover would send *K* = *H*(*W*) and *ω* encrypted with a symmetric key *K*_*PV*_ between prover and verifier. So no one can get the proof and the secret from the proof, except for the designated-verifier. At the same time, the verifier cannot obtain secret information except proof. We send *K* = *H*(*W*) and *ω* with encryption in security communication for resisting the replay attack and man-in-the-middle attack.

## Publicly-verifiable non-interactive zero-knowledge proof based on RLWE key exchange

### Syntax

**Fig 3:**
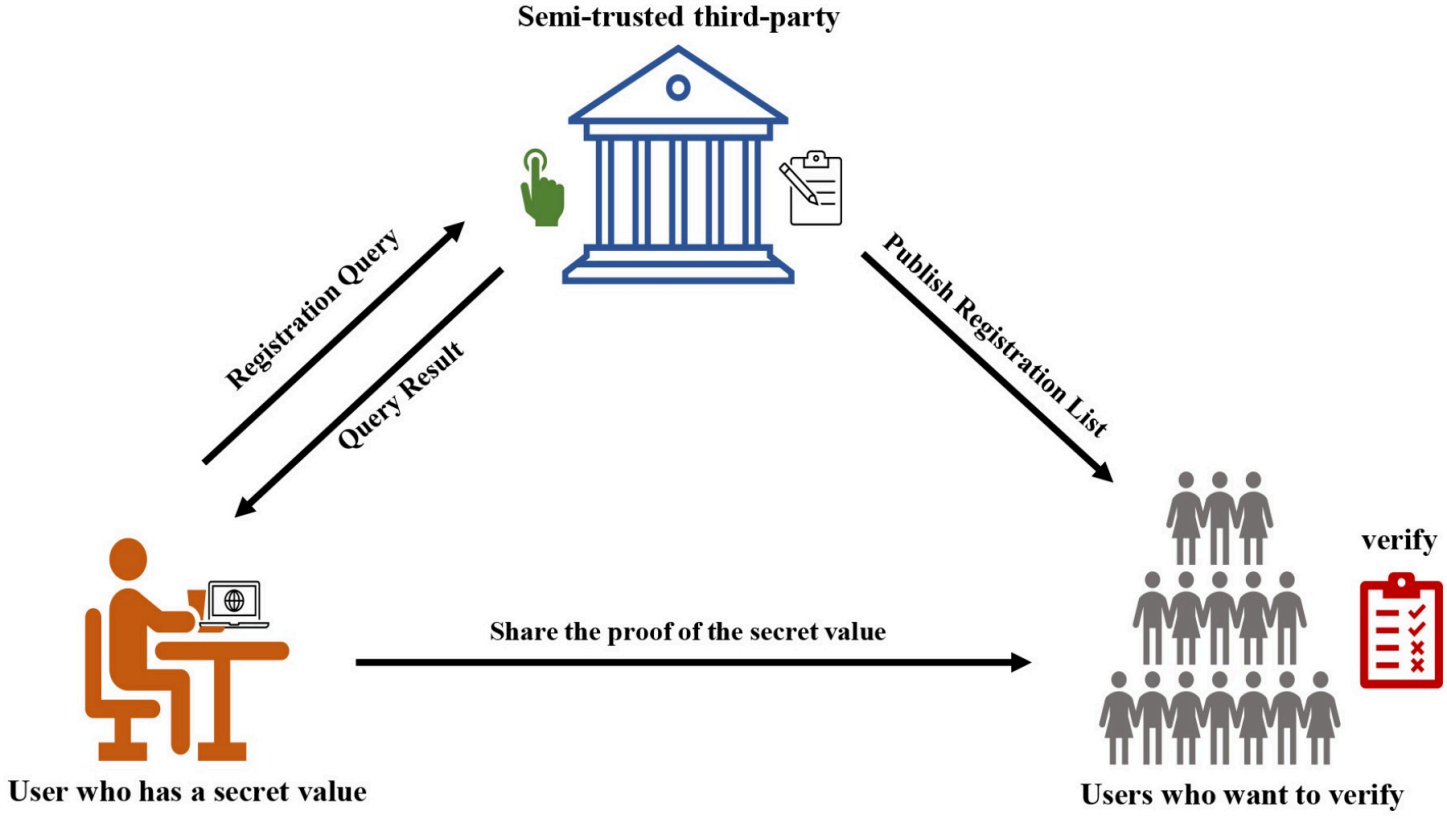
The publicly-verifiable scheme architecture.

We consider zero-knowledge proof in publicly-verifiable scenario for ownership with three participants: the third-party(T) who is curious-but-honest, prover(P) who provides the proof without revealing the secret, verifier(V) who verifies the prove in Fig 3.

**Fig 4:**
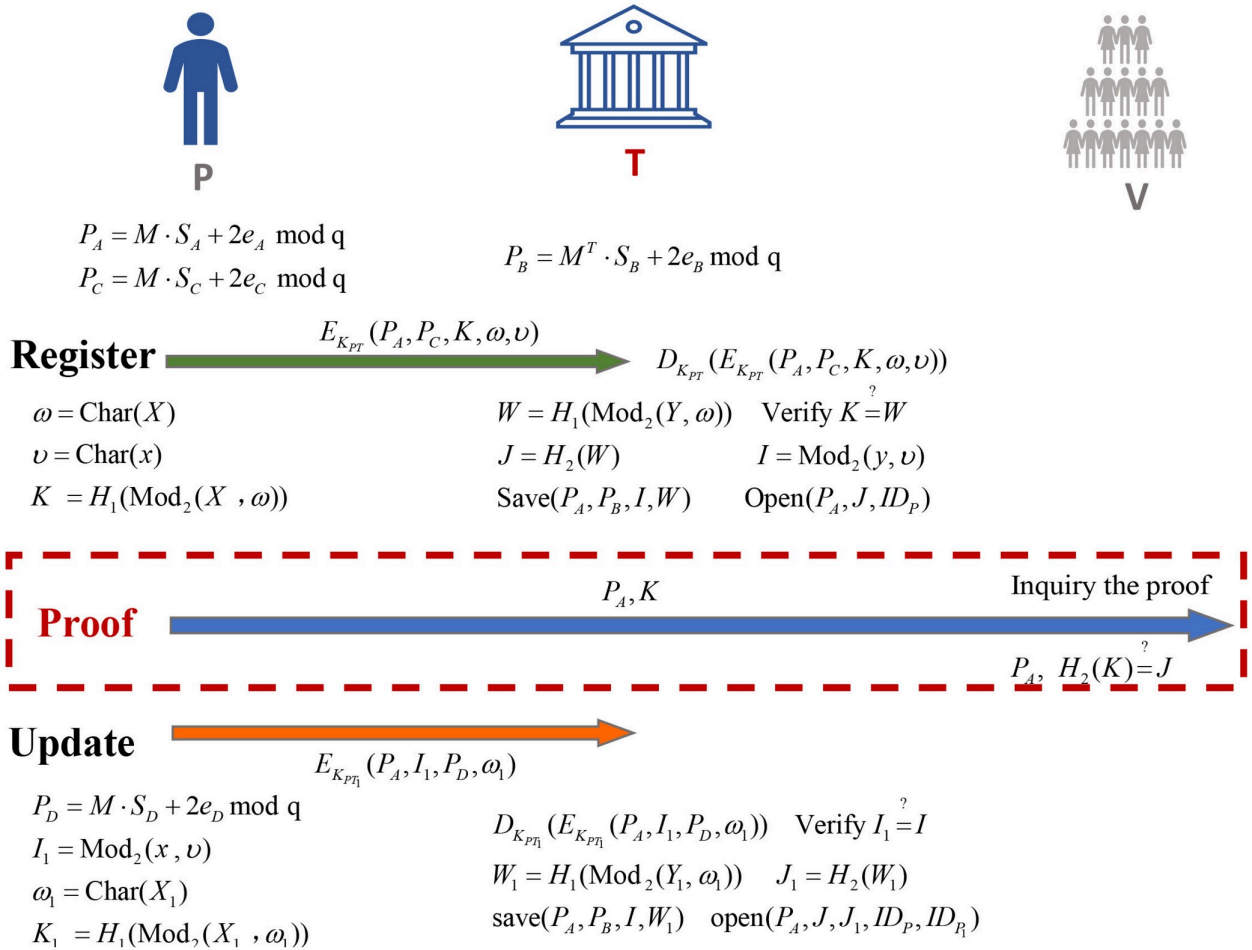
Publicly-verifiable non-interactive zero-knowledge based on key exchange.

A non-interactive zero-knowledge scheme based key exchange from lattice includes a set of five polynomial-time algorithms(Setup, Register, Proof, Verify, Update). Then we briefly describe the process followed by five polynomial-time algorithms, shown in Fig 4.

1. **Setup** (1^*p*^) → *P*: The setup algorithms takes as the security parameters *p*, everyone could choose uniformly random matrix *M* and obtain the secret vector *S* which is satisfying Discrete Gaussian Distribution, and outputs the public key *P*.
2. **Register** (*M*, *P*_*A*_, *P*_*C*_)→(*I*, *J*): The register algorithm takes as input the parameters *M*, public key of P is *P*_*A*_, public key of T for P is *P*_*B*_, the corresponding public key *P*_*C*_, signal function *ω* and *υ* for eliminating the errors, and outputs *I*, *W* and *J*. T would save *P*_*A*_, *I*, *W* and open *P*_*A*_, *J* and *ID*_*P*_ which is the identity of prover.
3. **Proof** (*S*_*A*_, *P*_*B*_, *P*_*C*_, *ω*) → *K*: Given *S*_*A*_, *P*_*B*_, *P*_*C*_, *ω*, P would obtain the zero-knowledge proof *K*. Then P should send the public key *P*_*A*_ and the zero-knowledge proof *K* to verifier without encryption.
4. **Verify** (*K*)→{0, 1}: After receiving the proof, V would find *J* by *P*_*A*_ from the third-party. Then V would verify if *H*(*K*) is equal to *J*.
5. **Update***J* → *J*, *J*_1_: Given the *P*_*A*_, *I*_1_, *P*_*D*_, *ω*_1_, T should verify whether *I*_1_ is equal to *I* or not firstly. If they were equal, then T would open *P*_*A*_, *J*, *J*_1_, *ID*_*P*_ and $ID_{P_{1}}$.

### Security model

In this subsection, we provide the security model for the publicly-verifiable non-interactive zero-knowledge proof Although the definition of non-interactive zero-knowledge remains unchanged, a semi-trusted third-party is introduced. Our security model is based on the curious-but-honest model and a random oracle model in federated learning [36, 43]. The semi-trusted third-party is curious-but-honest which means it does not deviate from the defined but tends to obtain all possible information from the legitimately received messages. In other words, the semi-trusted third-party only attempts to know the secret key of the prover, but he will not deceive other users with the proof he knows. Therefore, we can divide potential adversaries into the following:

1. An external adversary who is able to obtain public information. He attempts to give valid proof before the semi-trusted party issue.
2. The semi-trusted party as an internal adversary not only obtain the information from the phase of Register and Proof, but also possess the update key for all signers in the system.

In our second scheme, for the prover, he cannot forge an effective proof to deceive the semi-trusted third party and verifiers. For a semi-trusted third-party, he cannot obtain the secret key of the prover. For the verifier, he can neither obtain the secret key nor forge the proof of the secret value. Through the above analysis, it is clear that the security can be guaranteed if and only if the scheme satisfies the definition of zero-knowledge.

### The scheme in detail

The system first generates the public parameters *q*, *n*, *α*, where *q* > 8 is an odd prime. Let *M* be a uniformly random matrix $M\leftarrow R_{q}^{n\times n}$. Let *H*_1_: {0, 1}^*n*×*n*^ → {0, 1}^*n*^ and *H*_2_: {0, 1}^*n*×*n*^ → {0, 1}^*n*^ be random oracles. The secret information owner P has *P*_*A*_ = *M* ⋅ *S*_*A*_ + 2*e*_*A*_ mod *q* where *S*_*A*_, *e*_*A*_ ← *χ*_*α*_ are *n* × *n* dimensional same as above mentioned and *S*_*A*_ is secret information. Meanwhile, its public key is *P*_*C*_ = *M* ⋅ *S*_*C*_ + 2*e*_*C*_ mod *q* where *S*_*C*_, *e*_*C*_ ← *χ*_*α*_ are *n* × *n* dimensional and *S*_*B*_ is its secret key. Similarly for the third-party T, its public key for P is *P*_*B*_ = *M*^*T*^ ⋅ *S*_*B*_ + 2*e*_*B*_ mod *q* while *S*_*B*_ is its secret key. *P*_*A*_, *S*_*A*_, *P*_*B*_, *S*_*B*_, *P*_*C*_, *S*_*C*_ are used to create the zero-knowledge proof for verifiers V. Otherwise, T has a list for public and a symmetric key *K*_*PT*_ between prover and verifier.

The second scheme is composed of four algorithms except Setup algorithm: Register, Proof, Verify and Update. We specify it in full detail:

1.Register:

The secret information owner P does the following:

- Let $X=S_{A}^{T}\cdot P_{B}\cdot P_{C},\;x=S_{A}^{T}\cdot P_{B}$, P computes $C\leftarrow E_{K_{PT}}\left(P_{A},P_{C},K,\omega ,\upsilon \right)$, where *ω* = Char(*x*) and *υ* = Char(*x*).
- P sends *C* to T.

Upon receiving *C*, T does the following:

- T computes $\left(P_{A},P_{C},K,\omega ,\upsilon \right)\leftarrow D_{K_{PT}}\left(C\right)$.
- T checks if *P*_*A*_ ∈ list, reject. If *P*_*A*_ ∉ list and P is a certified legal user by T, T adds it to the list.
- Let $Y=P_{A}^{T}\cdot S_{B}\cdot P_{C},\;y=P_{A}^{T}\cdot S_{B}$, T computes *I* ← Mod_2_(*y*, *υ*) and *W* ← *H*_1_(Mod_2_(*Y*, *ω*)).
- T computes *J* ← *H*_2_(*W*).
- T saves (*P*_*A*_, *I*, *W*) and opens (*P*_*A*_, *P*_*B*_, *J*, *ID*_*P*_) Where *ID*_*P*_ is the identity of prover.

2.Proof:

- T computes *K* ← *H*_1_(Mod_2_(*X*, *ω*)) as the zero-knowledge proof. In fact, Mod_2_(*X*, *ω*) could be computed in the Register algorithm. The Proof algorithm only needs to calculate a hash operation.
- T opens (*P*_*A*_, *K*).

3.Verify:

Upon receiving (*P*_*A*_, *K*, *J*), everyone could do the following especially verifiers:

- After checking information is issued by *ID*_*P*_, then it computes *H*_2_(*K*) firstly.
- V checks if $H_{2}\left(K\right)\overset{?}{=}J$. Accept if *H*_2_(*K*) = *J*, otherwise reject.

4.Update:

The secret information owner *P*_1_ does the following:

- P computes $I^{{}^{\prime }}\leftarrow {\text{Mod}}_{2}\left(x,\upsilon \right)$.
- P samples *S*_*D*_, *e*_*D*_ ← *χ*_*α*_ and computes *P*_*D*_ = *M* ⋅ *S*_*D*_ + 2*e*_*D*_ mod *q*.
- Let $X_{1}=S_{A}^{T}\cdot P_{B}\cdot P_{D}$, P computes *ω*_1_ = Char(*X*_1_) and $C_{1}\leftarrow E_{K_{PT}}\left(P_{A},I^{{}^{\prime }},P_{D},\omega_{1}\right)$.
- P sends *C*_1_ to T.

Upon receiving *C*_1_, T does the following:

- T computes $\left(P_{A},I^{{}^{\prime }},P_{D},\omega_{1}\right)\leftarrow D_{K_{PT}}\left(C_{1}\right)$.
- Let $Y_{1}=P_{A}^{T}\cdot S_{B}\cdot P_{D}$, T checks if *I*′ is equal to *I*. If they are equal, then it outputs *W*_1_ ← *H*_1_(Mod_2_(*Y*_1_, *ω*)) and *J*_1_ ← *H*_2_(*W*_1_).
- T saves (*P*_*A*_, *I*, *W*, *W*_1_) and opens $\left(P_{A},P_{B},J,J_{1},ID_{P},ID_{P_{1}}\right)$.

### Security analysis

In this subsection, we prove that our zero-knowledge proof-based key exchange scheme is security over LWE in public under the hardness assumption from the following aspects.

**Lemma 7***Let*$R=Z\left[x\right]/\left(x^{n}+1\right)$, *for anya*, *b*, *c* ∈ *R*, *we can easily obtain* ‖*a* ⋅ *b* ⋅ *c*‖ ≤ *n* ⋅ ‖*a*‖ ⋅ ‖*b*‖ ⋅ ‖*c*‖ and ‖*a* ⋅ *b* ⋅ *c*‖_∞_ ≤ ‖*a*‖_∞_ ⋅ ‖*b*‖_∞_ ⋅ ‖*c*‖_∞_ *by Lemma 1*.

**Lemma 8***Let q* > 16*σ*^3^ *n*^*α*5/2^, *then* Mod_2_(*X*, *ω*) = Mod_2_(*Y*, *ω*), *except with negligible probability*.

**Proof 0.5***Firstly, we know that*$X=S_{A}^{T}\cdot P_{B}\cdot P_{C}\;\;,\;\;Y=P_{A}^{T}\cdot S_{B}\cdot P_{C}$.

$$\begin{array}{c} Then,X-Y=\left(x-y\right)\cdot P_{C} \end{array}$$

*Prove similarly to Lemma 4*, $X,Y\in R_{q}^{n\times n}$, *then we just show that*$\|X_{i,j}-Y_{i,j}{\|}_{\infty }<\frac{q}{4}$*by Lemma 3, where i*, *j* ∈ {1, ⋯, *q*}. *After simplification, combining with Lemma 2 and Lemma 7, we have that*

$$\begin{array}{cc} \|X_{i,j}-Y_{i,j}\| & \leq \|2\left[S_{A_{i,j}}^{T}\cdot e_{B_{i,j}}-e_{A_{i,j}}^{T}\cdot S_{B_{i,j}}\right]\|\cdot \|P_{C}\|\\ & <4n\cdot {\left(\sigma \sqrt{n}\right)}^{3} \end{array}$$

*By Lemma 3, we obtain* $\|X_{i,j}-Y_{i,j}{\|}_{\infty }<4\sigma^{3}n^{5/2}<\frac{q}{4}$.

*Then, q* > 16*σ*^3^ *n*^5/2^ *with probability* 1 − 2^−*n*^.

#### 1) Completeness

The proof from prover is as follows:

Since *K* = *H*_1_(Mod_2_(*X*, *ω*)), Mod_2_(*X*, *ω*) could be calculated in the previous Register algorithm, then *K* is only obtained by the hash operation as the proof for verifier.

The verifier gets the verification value *J* from the third-party and verifies *K* whether it is true or not by the hash function *J* = *H*_2_(*K*).

By Lemma 8, let *q* > 16*σ*^3^ *n*^*α*5/2^, if $S_{A}^{T}$ is true, then

$${\text{Mod}}_{2}\left(X,\omega \right)={\text{Mod}}_{2}\left(Y,\omega \right).$$

except with negligible probability.

Then, we have that

$$K=H_{1}({\text{Mod}}_{2}\left(X,\omega \right))=H_{1}({\text{Mod}}_{2}\left(Y,\omega \right))=W$$

Finally, if *K* is true, *H*_2_(*K*) will be equal to *J* with probability 1 − 2^−*n*^, where *J* = *H*_2_(*W*). Then the verifier will be convinced that the prover owns the secret. That means, for $\forall P_{A},P_{B},P_{C}\in L$, if the prover has the secret value $S_{A}^{T}$, then

$$Pr\;\left[K\leftarrow P\left(S_{A}^{T},P_{A},P_{B},P_{C}\right):V\left(J_{\left(P_{A},P_{B},P_{C}\right)},K\right)=1\right]\geq 1-2^{-n}.$$

#### 2) Soundness

Soundness means that the adversary has no secret value *S*_*A*_ and convinces the honest verifier that the secret value is true with negligible probability. In other words, if the prover has no secret value *S*_*A*_, for ∃*P*_*A*_, *P*_*B*_, *P*_*C*_, then

$$Pr\;[K^{{}^{\prime }}\leftarrow P\left(P_{A},P_{B},P_{C}\right):V\left(J_{\left(P_{A},P_{B},P_{C}\right)},K^{{}^{\prime }}\right)=1]=\text{negl}.$$

Its analysis is similar to the soundness of the designated-verifier scheme in Fig 1. According to the second scheme in Fig 2, there are three participants: the third-party(T) who is curious-but-honest, prover(P), and verifier(V). Then we should consider this property from the third-party and the verifier.

- Between a malicious prover and the third-party.

Assume prover as the adversary that can register without the secret *S*_*A*_ with the non-negligible probability. This analysis is similar to the soundness of the designated-verifier scheme in Fig 1. According to Lemma 5, we can get the following Lemma 9 easily. Then the adversary cannot register successfully the verifier to believe him without knowing the secret value with negligible probability.

**Lemma 9***If prover as the adversary*A*who could register successfully usingP*_*A*_ = *M* ⋅ *S*_*A*_ + 2*e*_*A*_ *mod* *q* *without the secret of S*_*A*_ *in the curious-but-honest third-party with non-negligible probability, then there is a distinguisher for P*_*A*_ *from uniform with the same probability*.

- Between a malicious prover and the verifiers.

Assume prover as the adversary that can make a valid proof *K* without the secret *S*_*A*_ with the non-negligible probability. According to the second scheme in Fig 2, the adversary only gets valid proof from *P*_*A*_ or *J*. Since the analysis of the above, we have already known that the prover is the adversary who only has *P*_*A*_ without *S*_*A*_ to convince the verifiers with negligible probability. Then the malicious prover only makes a valid proof from *J*. As the scheme described in Fig 2, we know that *J* = *H*_2_(*K*). That means the adversary can calculate the inverse of the hash function output *J*. However, one of the important hash function properties is that if *H* is collision-resistant, then *H*(*x*) is hard to invert.

**Lemma 10***If prover as the adversary*A*who could who could generate a valid proof K* for *P*_*A*_ = *M* ⋅ *S*_*A*_ + 2*e*_*A*_ *mod* *q* *without the secret of S*_*A*_ *with non-negligible probability, then there is a distinguisher for* *P*_*A*_ *from uniform with the same probability, or the adversary can calculate the inverse of the hash function output*.

Above all considering, we believe that the adversary cannot successfully deceive the verifier without *S*_*A*_, then we have that

$$Pr\;[K\leftarrow Adversary\left(,P_{A},P_{B},P_{C}\right):V\left(J_{\left(P_{A},P_{B},P_{C}\right)},K\right)=1]=\text{negl}.$$

#### 3) Zero-knowledge

In the second scheme, *S*_*A*_ as the secret value, it must not be a leak in the whole process of proof. Because the proof can be verified by anyone especially the verifier, so we consider this property from the verifier and the third-party.

- For the verifier:

From the scheme beginning to the end, the verifier can only obtain *P*_*A*_, *K*, *J*, where

$$\begin{array}{c} P_{A}=M\cdot S_{A}+2e_{A}\;\;\text{mod}\;q \end{array}$$ (1)

$$\begin{array}{c} K=H_{1}\left({\text{Mod}}_{2}\left(X,\omega \right)\right) \end{array}$$ (2)

$$\begin{array}{c} J=H_{2}\left(W\right)=H_{2}\left(H_{1}\left({\text{Mod}}_{2}\left(Y,\omega \right)\right)\right) \end{array}$$ (3)

From Eq (1), we can see that *S*_*A*_ will not be obtained from *P*_*A*_ under the Ring-LWE assumption. Since *K* and *J* are the results of hash function operation from equations Eqs (2) and (3), where *K* and *J* are *n* bits value, the secret value *S*_*A*_ will not be disclosed. According to the above analysis of soundness, the verifier can not get the secret value or a valid proof.

- For the third-party:

T, who is curious-but-honest, acts as the intermediary between the prover and the verifier. T has more values including *S*_*B*_, *P*_*B*_, *ω*, *υ*, *I*, *W* except *S*_*A*_.

$$\begin{array}{c} \omega =\text{Char}(X)\;\;\upsilon =\text{Char}(x) \end{array}$$ (4)

$$\begin{array}{c} I={\text{Mod}}_{2}\left(y\;,\;\upsilon \right) \end{array}$$ (5)

$$\begin{array}{c} W=H_{1}\left({\text{Mod}}_{2}\left(Y,\omega \right)\right) \end{array}$$ (6)

In fact, the third-party has known the proof before the prover announced it. T is curious-but-honest means that T is eager to know the secret value of *S*_*A*_ and can not reveal any other values. From equations Eqs (4)–(6), we can make our second scheme is zero-knowledge for the third-party according to the above Lemma 6. Then this scheme is the honest-verifier zero-knowledge for the third-party.

Based on the above discussion, we can get the conclusion that there is no secret value leakage. Assume that there is a polynomial-time simulator, for all *P*_*A*_, *P*_*B*_, *P*_*C*_ ∈ *L* and secret value *S*_*A*_, the following two distributions are computationally indistinguishable

$$\|Pr\;\left[Exp_{ZK-real}=1\right]-Pr\;\left[Exp_{ZK-sim}=1\right]\;\|=\text{negl}.$$

The prover sends *K* with encryption in security communication for resisting the brute force attack and man-in-the-middle attack. In order to resist the replay attacks, the third-party opens *ID*_*P*_ except *P*_*A*_, *J*.

## Efficiency analysis

According to existing post-quantum schemes, we analyze the efficiency of the zero-knowledge proof schemes from the prover runtime, verification time, CRS size, and proof size [44]. Since the proof can be given off-line in advance, the prover’s operation time is no longer considered here. Meanwhile, our schemes based on lattice key exchange do not involve CRS, so the size of CRS can be considered to be zero. Therefore, our proposed scheme is only compared with the previous study [27, 31–35] in verification time and proof size, which is described in more detail in Table 2.

**Table 2:** Comparison the efficiency of related zero-knowledge proof schemes.

Scheme	Proof Size	Verification Time	Interactive	Untrusted-Setup	Assumptions
[27]	4*nMlog*(*q*)	*ploy*(*n*)	YES	YES	RLWE
[31]	$\sqrt{mlog(m)}$	*m*	YES	YES	SIS
[32]	1024*t*	*ploy*(*n*)	NO	YES	RSIS & RLWE
[33]	*c*_2_ *n*_2_	*ploy*(*n*)	YES	YES	LWE
[34]	512 + *c*_1_ *n*_1_	*ploy*(*n*)	NO	YES	SVP
[35]	*k*_1_	*l*_1_	NO	NO	q-PKE
Our scheme I	*k*_2_	*ploy*(*n*)	NO	YES	RLWE
Our scheme II	*k*_2_	*l*_2_	NO	SEMI	RLWE

In previous studies, the proof size in [27] is more than 4*nMlog*(*q*) bits, where *n* is a security parameter in the ring $Z_{q}$ and *M* means the number of protocol rounds. And the *ploy*(*n*) means that the verification time is polynomial-time which is used in the following. In [31], the proof size is $\sqrt{mlog(m)}$ and the verification time is *m*, where *m* is the number of gates. In [32], the proof size is more than 1024*t* bits, where *t* means repeating the protocol times. The proof size in [34] is larger than 512 + *n*_1_(8log(4*αn*_1_) + 2048log(2*α*))bits, where *n*_1_ is the number of secrets, *α* is the security parameter. In Table 1, *c*_1_ is used to represent a number greater than 8log(4*αn*_1_) + 2048log(2*α*). The proof size and the verification time in [35] are constant. Here, *k*_1_ = 5(*a* + 1)log(*q*) represents the proof size, where *a* is the number of elements in the vector, which is also a security parameter. And *l*_1_ represents the verification time. However, this scheme is based on ZK-SNARKs from SSP, the proof size is still large and the cost of computation is relatively expensive. At this time, the CRS size is more than (3*d* + 2)log(*q*), where *d* is the degree of SSP. The proof size in [33] is *n*_2_ ⋅ *k* ⋅ log(6*σ*) + *n*_2_ ⋅ ℓ ⋅ log(*q*) which is replaced by *c*_2_ *n*_2_. Where *n*_2_ and *q* are security parameters, *n*_2_ is also the bit number of secrets, *k* is the width of the commitment matrices over *R*_*q*_, *σ* is the standard deviation. The commitments are a guarantee condition sent by the prover to the verifier in commitment scheme [33]. when the width of the commitment matrices over *R*_*q*_ is equal to 1, the commitment size is *n*_2_ ⋅ log(*q*). Our schemes combine the key-exchange based on RLWE with the hash function so that proof size is only the output length of the hash function. *k*_2_ represents the proof size of our schemes. Verification time is expressed as *l*_2_, which is a hash function operation for the publicly-verifiable scenario. Furthermore, Table 2 shows a comparison of other properties, including CRS or interactive, untrusted-Setup and assumptions, where q-PKE is the q-power knowledge of exponent assumption, (R)SIS is (Ring) short integer solution problem and SVP means shortest vector problem.

**Fig 5:**
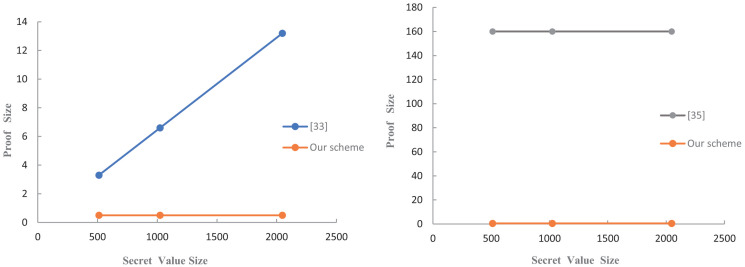
Impact of the secret value size on proof size.

As shown in the result, our proof is the output of the hash function which its size does not change due to the size of secret values. In other words, the proof size does not become as large as the size of secret values increases. The commitment will be sent to the verifier along with the proof, as a condition for verification like a CRS. Therefore, we also give the corresponding commitment size in [33] and the CRS size in [35]. Given the security parameter is 1024, *q* is 2^32^, *d* is 2^15^, and the fixed output of the hash function is 512 bits. To intuitively show the performance of our second scheme, we compared the proof size of our second scheme, the optimal scheme of Baum et al. [33], and the scheme of [35] in Table 3 and in Fig 5. And the result indicates that our second scheme proof size is much smaller and constant.

**Table 3:** Approximate comparison.

Scheme	Other	Proof Size
Our scheme II	0	0.5KB
[33]	Commitment Size ≈ 8.1KB	6.6KB
[35]	CRS Size ≈ 3MB	160KB

For the verification time, our second scheme has only one hash operation time. However, the scheme of Baum et al. takes polynomial time to verify a matrix polynomial. For the scheme of [35], the verifier needs to encode several polynomials before calculating three polynomials during verification. Obviously, both of the above schemes take more time than our second scheme.

We have compared our second scheme with the related schemes in zero-knowledge proof size and verification time. Since the proof is the output of the hash function, our proof size is constant. So its size is smaller than that of other compared schemes in zero-knowledge proof size. In addition, the verifiers’ runtime of our scheme for publicly-verifiable only involves the hash operation, so its computation is lower than that of other compared schemes in verification time.

## Conclusion

With the surprising development of quantum computers, it is an urgent requirement to construct efficient quantum-secure zero-knowledge proof schemes. In this paper, we have proposed a non-interactive zero-knowledge proof scheme for the designated-verifier to guarantee less proof size. We have also designed the scheme of RLWE-based key exchange from lattice for the publicly-verifiable scenario to ensure better effectiveness. Moreover, our schemes are secure from completeness, soundness, and zero-knowledge. Furthermore, compared with other previous schemes, we find that our schemes have more advantages in proof size and verification time. In the future, based on the abundant theoretical basis of lattice cryptography, we will design better performed zero-knowledge proof schemes for multiple applications.

## Supporting information

S1 Table The process of approximate comparison. (XLSX)

S1 Fig Impact of the secret value size on proof size. (XLSX)
